# Genome Sequence of *Pantoea* sp. Strain 1.19, Isolated from Rice Rhizosphere, with the Capacity To Promote Growth of Legumes and Nonlegumes

**DOI:** 10.1128/genomeA.00707-17

**Published:** 2017-07-27

**Authors:** Esaú Megías, Fábio Bueno Reis Junior, Renan Augusto Ribeiro, Manuel Megías, Francisco Javier Ollero, Mariangela Hungria

**Affiliations:** a Universidad de Sevilla, Facultad de Biología, Departamento de Microbiología, Sevilla, Spain; b Embrapa Cerrados, Soil Microbiology, Planaltina, DF, Brazil; c CNPq, Lago Sul, Brasília, Federal District, Brazil; d Embrapa Soja, Soil Biotechnology, Londrina, Paraná, Brazil

## Abstract

*Pantoea* sp. 1.19, a plant growth-promoting bacterium (PGPB), was isolated from the rhizosphere of rice plants in Spain. Its genome, estimated at 3,771,065 bp, encodes 3,535 coding sequences (CDSs), carrying genes for synthesis of auxins, homoserine lactones, enzymes, siderophores, and quorum sensing. Several CDSs emphasize its biotechnological potential as an agriculture inoculant.

## GENOME ANNOUNCEMENT

Agricultural sustainability relies greatly on an adequate equilibrium of soil microbes (1), and inoculants containing one or more elite bacterial species have been increasingly used, improving crops yields (2). Our group has isolated *Pantoea* spp. in rice paddies of the Guadalquivir River marshes, southern Spain, from both the rhizosphere and as rice endophytes. Previously we sequenced the genome of the endophytic *Pantoea ananatis* strain AMG521 (3) and now we present the genome of *Pantoea* sp. strain 1.19, isolated from the rice rhizosphere. 1.19 is a plant growth-promoting bacterium (PGPB) showing outstanding properties such as production of siderophores, auxins (indole acetic acid-IAA, 56 mg mL^−1^), ACC (1-aminocyclopropane-1-carboxylate) deaminase, and amilase. The AHLs (N-acyl-homoserine-lactones) synthesized by 1.19 were identified as C6-AHL and 3-oxo-C6 AHL. Rhizospheric and foliar application of 1.19 increases plant biomass and/or grain production—by 10 to 50%—of legumes (alfafa, *Medicago sativa*), pastures (*Urochloa brizantha*), and cereals (rice, *Oriza sativa*).

To access the bacterial genome, total DNA was extracted using the DNeasy blood and tissue kit (Qiagen) and processed on the MiSeq plataform (Illumina) at Embrapa Soja. Paired-end reads obtained by shotgun sequencing allowed a genome coverage of 65-fold. The FASTQ files were assembled by the A5-MiSeq pipeline (*de novo* assembly) (4). The genome was estimated at 3,792,740 bp, assembled in 41 contigs, with a G+C content of 59.9 mol%. Sequences were submitted to RAST (5) and the annotation identified 3,535 CDSs, and 58% were classified in 507 subsystems. The major categories were carbohydrates (12.9%), amino acids and derivatives (12.6%), proteins (8.3%), cofactors, vitamins, prosthetic groups, pigments (8.2%), and RNA metabolism (7.5%).

At least 17 carbohydrate-related genes similar to those described for *Pantoea vagas* and *P. ananatis* (6) were found in the genome of 1.19. Genes of types I, II, III, IV, V, VI, and VII secretion systems and of biosynthesis of siderophores (aerobactin in the outer membrane and alcaligin-like types) were found. In relation to quorum sensing, AHL-related genes (*yspI*/*yspR*) are present; however, they are different from the *luxI*/*luxR* homologous described in *P. ananatis* LMO 2665^T^ (*eanI*/*eanR* and *rhlI*/*rhlR*), both required for biofilm formation that confers pathogenicity to this strain (7). This is an indication of the lack of pathogenicity of 1.19. We found five genes related to IAA, as well as to the degradation of salicylate, two important properties of PGPB. A large number of genes (5.3%) are related to stress response, including oxidative stress, cold and heat shock, choline, betaine, and trehalose biosynthesis. The genome also carries genes for the biosynthesis of acetoin and butanediol. All these properties highlight the biotechnological potential of *Pantoea* sp. 1.19. Noteworthy is the low similarity of the genome of 1.19 with all described related species, with average nucleotide identity (ANI) lower than 80% with *Pantoea* and with the related genera *Erwinia* and *Tatumella*, indicating that it might represent a new taxa.

### Accession number(s).

This whole-genome shotgun project has been deposited at DDBJ/EMBL/GenBank under the SUBID SUB2154307, BioProject PRJNA356250, BioSample SAMN06111205, accession no. MRBS00000000.
